# Supplementary material for: Theoretical Study of Copper Squarate as a Promising Adsorbent for Small Gases Pollutants
Source: Molecules. 2024 Jul 2;29(13):3140. doi: 10.3390/molecules29133140 (PMC11243752; doi:10.3390/molecules29133140)
Supplement: Supplementary file 1 [file molecules-29-03140-s001.zip › molecules-3057836-supplementary.pdf]

SUPPLEMENTARY MATERIAL

Theoretical Study of Copper Squarate as a Promising Adsorbent for Small Gases Pollutants

Celia Adjal [1,2], Nabila Guechtouli [1,3], Vicente Timón [2,*] , Francisco Colmenero [4] and Dalila Hammoutène [1]

1 Laboratory of Thermodynamics and Molecular Modeling, Faculty of Chemistry, University of Science and Technology Houari Boumediene (USTHB), BP32, El Alia, Bab Ezzouar, Algiers 16111, Algeria; adjalcelia5@gmail.com (C.A.); n.guechtouli@univ-boumerdes.dz (N.G.); dhammoutene@yahoo.fr (D.H.)

2 Instituto de Estructura de la Materia, CSIC, Serrano 121, 28006 Madrid, Spain

3 Faculty of Sciences, University of M'hamed Bougara, (UMBB), Boumerdes 35000, Algeria

4 Centro de Investigaciones Energéticas, Medioambientales y Tecnológicas (CIEMAT), Avda/Complutense, 40, 28040 Madrid, Spain; francolm@ucm.es

* Correspondence: vicente.timon@csic.es

Figure S1: Comparison between the Powder X-ray diffraction patterns derived from the computed and experimental structures*.

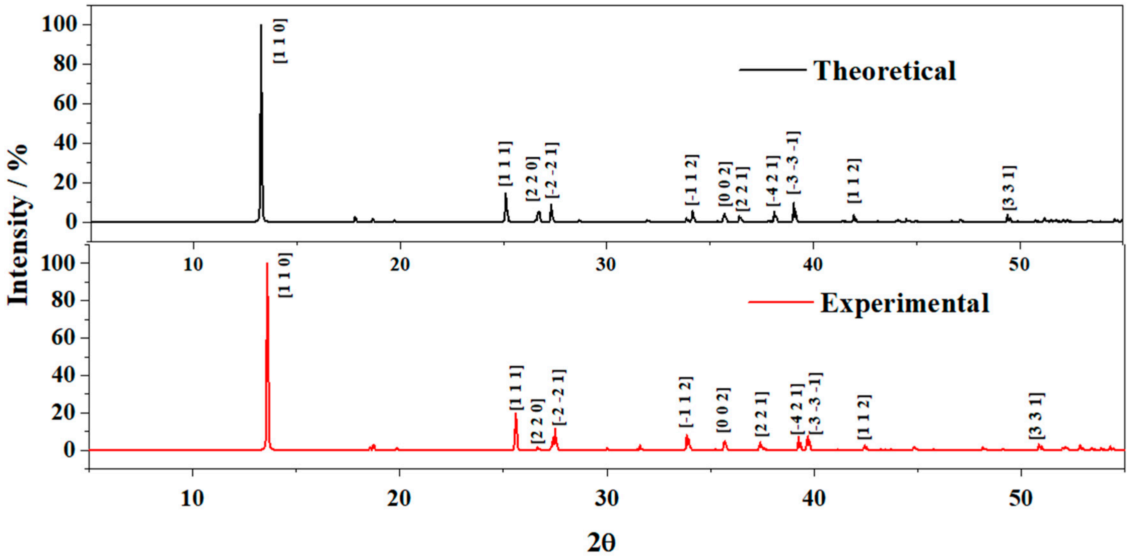

* Dinnebier, R.E.; Nuss, H.; Jansen, M. Anhydrous CuC_4O_4, a Channel Structure Solved from X-Ray Powder Diffraction Data. Z. Anorg. Allg. Chem. 2005, 631, 2328–2332, doi:10.1002/zaac.200500232.

Table S1: Comparison between the theoretical data on the geometry of molecules and the experimental data obtained from NIST database.

Molecules	Bond distances (Å)			Angles (˚)		
	Bond	Theor.	Exp*.	Angle	Theor.	Exp*.
H_2O	OH	0.962	0.957	HOH	105.669	104.477
CH_4	CH	1.092	1.087	HCH	109.357	109.471
CO_2	CO	1.163	1.162	OCO	179.986	180
SO_2	SO	1.438	1.432	OSO	118.499	119.57
N_2O	NN<br>NO	1.141<br>1.179	1.128<br>1.184	NNO	179.686	180
NF_3	NF	1.404	1.365	FNF	102.073	102.37
CF_4	CF	1.341	1.315	FCF	109.314	109.47
SF_6	SF	1.585	1.561	FSF	89.859	90
0_3	OO	1.267	1.278	OOO	117.303	116.8

*NIST Computational Chemistry Comparison and Benchmark Database
NIST Standard Reference Database Number 101
Release 22, May 2022, Editor: Russell D. Johnson III
http://cccbdb.nist.gov/
DOI:10.18434/T47C7Z

Table S2: The reported calculated structure in *.cif format, used in the DFT-PBE calculations

**Copper squarate double cell**
data_Copper-squarate<br>_audit_creation_date 2023-05-09<br>_audit_creation_method 'Materials Studio'<br>_symmetry_space_group_name_H-M 'P1'<br>_symmetry_Int_Tables_number 1<br>_symmetry_cell_setting triclinic<br>loop_<br>_symmetry_equiv_pos_as_xyz<br>x,y,z<br>_cell_length_a 11.0171<br>_cell_length_b 9.3521<br>_cell_length_c 11.2885<br>_cell_angle_alpha 89.9183<br>_cell_angle_beta 117.6359<br>_cell_angle_gamma 90.0822<br>loop_<br>_atom_site_label<br>_atom_site_type_symbol<br>_atom_site_fract_x<br>_atom_site_fract_y<br>_atom_site_fract_z<br>_atom_site_U_iso_or_equiv<br>_atom_site_adp_type<br>_atom_site_occupancy<br>O1 O 0.12049 0.25620 0.11101 0.00000 Uiso<br>1.00<br>C2 C 0.19010 0.25550 0.05068 0.00000 Uiso<br>1.00<br>C3 C 0.30555 0.16469 0.06203 0.00000 Uiso<br>1.00<br>O4 O 0.36949 0.06438 0.13916 0.00000 Uiso<br>1.00<br>O5 O 0.62116 0.75505 0.11150 0.00000 Uiso<br>1.00<br>C6 C 0.69012 0.75544 0.05046 0.00000 Uiso<br>1.00<br>C7 C 0.80529 0.66504 0.06172 0.00000 Uiso<br>1.00<br>O8 O 0.86891 0.56485 0.13922 0.00000 Uiso<br>1.00<br>O9 O 0.87906 0.25672 0.13865 0.00000 Uiso<br>1.00<br>C10 C 0.80984 0.25592 0.19943 0.00000 Uiso<br>1.00<br>C11 C 0.69448 0.16499 0.18850 0.00000 Uiso<br>1.00<br>O12 O 0.63054 0.06461 0.11168 0.00000 Uiso<br>1.00<br>O13 O 0.37914 0.75494 0.13871 0.00000 Uiso<br>1.00

C14	C	0.30986	0.75519	0.19945	0.00000	Uiso	1.00
C15	C	0.19451	0.66474	0.18834	0.00000	Uiso	1.00
O16	O	0.13059	0.56454	0.11115	0.00000	Uiso	1.00
O17	O	0.87881	0.74337	0.38845	0.00000	Uiso	1.00
C18	C	0.80976	0.74432	0.44935	0.00000	Uiso	1.00
C19	C	0.69456	0.83524	0.43828	0.00000	Uiso	1.00
O20	O	0.63042	0.93542	0.36111	0.00000	Uiso	1.00
O21	O	0.37818	0.24486	0.38778	0.00000	Uiso	1.00
C22	C	0.30948	0.24449	0.44909	0.00000	Uiso	1.00
C23	C	0.19433	0.33489	0.43805	0.00000	Uiso	1.00
O24	O	0.13040	0.43488	0.36047	0.00000	Uiso	1.00
O25	O	0.12035	0.74282	0.36111	0.00000	Uiso	1.00
C26	C	0.18978	0.74383	0.30054	0.00000	Uiso	1.00
C27	C	0.30515	0.83487	0.31160	0.00000	Uiso	1.00
O28	O	0.36916	0.93526	0.38846	0.00000	Uiso	1.00
O29	O	0.62079	0.24515	0.36159	0.00000	Uiso	1.00
C30	C	0.68992	0.24479	0.30069	0.00000	Uiso	1.00
C31	C	0.80528	0.33510	0.31174	0.00000	Uiso	1.00
O32	O	0.86918	0.43520	0.38906	0.00000	Uiso	1.00
Cu33	Cu	0.00042	0.41710	0.12619	0.00000	Uiso	1.00
Cu34	Cu	0.50140	0.91433	0.12729	0.00000	Uiso	1.00
Cu35	Cu	-0.00147	0.58253	0.37325	0.00000	Uiso	1.00
Cu36	Cu	0.49849	0.08567	0.37282	0.00000	Uiso	1.00
O37	O	0.12108	0.25678	0.61184	0.00000	Uiso	1.00
C38	C	0.18996	0.25591	0.55075	0.00000	Uiso	1.00
C39	C	0.30512	0.16501	0.56171	0.00000	Uiso	1.00
O40	O	0.36907	0.06474	0.63886	0.00000	Uiso	1.00
O41	O	0.62141	0.75608	0.61210	0.00000	Uiso	1.00

C42	C	0.69021	0.75601	0.55091	0.00000	Uiso	1.00
C43	C	0.80534	0.66538	0.56208	0.00000	Uiso	1.00
O44	O	0.86926	0.56536	0.63959	0.00000	Uiso	1.00
O45	O	0.87945	0.25727	0.63873	0.00000	Uiso	1.00
C46	C	0.81005	0.25625	0.69931	0.00000	Uiso	1.00
C47	C	0.69445	0.16532	0.68804	0.00000	Uiso	1.00
O48	O	0.63012	0.06528	0.61098	0.00000	Uiso	1.00
O49	O	0.37895	0.75497	0.63821	0.00000	Uiso	1.00
C50	C	0.31002	0.75528	0.69933	0.00000	Uiso	1.00
C51	C	0.19468	0.66495	0.68828	0.00000	Uiso	1.00
O52	O	0.13061	0.56496	0.61088	0.00000	Uiso	1.00
O53	O	0.87977	0.74329	0.88898	0.00000	Uiso	1.00
C54	C	0.81025	0.74414	0.94938	0.00000	Uiso	1.00
C55	C	0.69480	0.83505	0.93802	0.00000	Uiso	1.00
O56	O	0.63101	0.93533	0.86089	0.00000	Uiso	1.00
O57	O	0.37927	0.24429	0.88866	0.00000	Uiso	1.00
C58	C	0.31027	0.24412	0.94966	0.00000	Uiso	1.00
C59	C	0.19511	0.33466	0.93832	0.00000	Uiso	1.00
O60	O	0.13162	0.43488	0.86080	0.00000	Uiso	1.00
O61	O	0.12112	0.74328	0.86155	0.00000	Uiso	1.00
C62	C	0.19030	0.74404	0.80072	0.00000	Uiso	1.00
C63	C	0.30578	0.83492	0.81175	0.00000	Uiso	1.00
O64	O	0.37002	0.93507	0.88870	0.00000	Uiso	1.00
O65	O	0.62103	0.24492	0.86143	0.00000	Uiso	1.00
C66	C	0.69014	0.24483	0.80050	0.00000	Uiso	1.00
C67	C	0.80557	0.33521	0.81170	0.00000	Uiso	1.00
O68	O	0.86982	0.43520	0.88900	0.00000	Uiso	1.00
Cu69	Cu	0.00108	0.41764	0.62662	0.00000	Uiso	1.00

```
Cu70   Cu    0.49951   0.91469   0.62507   0.00000  Uiso
1.00
Cu71   Cu    0.00052   0.58258   0.87445   0.00000  Uiso
1.00
Cu72   Cu    0.49974   0.08541   0.87357   0.00000  Uiso
1.00
loop_
_geom_bond_atom_site_label_1
_geom_bond_atom_site_label_2
_geom_bond_distance
_geom_bond_site_symmetry_2
_ccdc_geom_bond_type
O1     C2      1.240    .     D
O1     Cu33    2.064    .     S
C2     C3      1.486    .     S
C2     C59     1.491    1_554 S
C3     O4      1.251    .     D
C3     C58     1.491    1_554 S
O4     Cu34    2.070    1_545 S
O5     C6      1.240    .     D
O5     Cu34    2.053    .     S
C6     C7      1.481    .     S
C6     C55     1.492    1_554 S
C7     O8      1.251    .     D
C7     C54     1.490    1_554 S
O8     Cu33    2.058    1_655 S
O9     C10     1.240    .     D
O9     Cu33    2.056    1_655 S
C10    C11     1.485    .     S
C10    C31     1.490    .     S
C11    O12     1.250    .     D
C11    C30     1.492    .     S
O12    Cu34    2.063    1_545 S
O13    C14     1.240    .     D
O13    Cu34    2.051    .     S
C14    C15     1.482    .     S
C14    C27     1.491    .     S
C15    O16     1.251    .     D
C15    C26     1.489    .     S
O16    Cu33    2.051    .     S
O17    C18     1.240    .     D
O17    Cu35    2.061    1_655 S
C18    C19     1.485    .     S
C18    C43     1.491    .     S
C19    O20     1.251    .     D
C19    C42     1.490    .     S
O20    Cu36    2.071    1_565 S
O21    C22     1.240    .     D
O21    Cu36    2.053    .     S
C22    C23     1.482    .     S
C22    C39     1.492    .     S
C23    O24     1.251    .     D
C23    C38     1.490    .     S
O24    Cu35    2.059    .     S
O25    C26     1.240    .     D
O25    Cu35    2.057    .     S
```

C26	C27	1.485	.	S
C27	O28	1.251	.	D
O28	Cu36	2.066	1_565	S
O29	C30	1.240	.	D
O29	Cu36	2.051	.	S
C30	C31	1.481	.	S
C31	O32	1.251	.	D
O32	Cu35	2.047	1_655	S
Cu33	O9	2.056	1_455	S
Cu33	O8	2.058	1_455	S
Cu34	O4	2.070	1_565	S
Cu34	O12	2.063	1_565	S
Cu35	O17	2.061	1_455	S
Cu35	O32	2.047	1_455	S
Cu36	O20	2.071	1_545	S
Cu36	O28	2.066	1_545	S
O37	C38	1.240	.	D
O37	Cu69	2.062	.	S
C38	C39	1.485	.	S
C39	O40	1.251	.	D
O40	Cu70	2.067	1_545	S
O41	C42	1.240	.	D
O41	Cu70	2.053	.	S
C42	C43	1.482	.	S
C43	O44	1.251	.	D
O44	Cu69	2.060	1_655	S
O45	C46	1.240	.	D
O45	Cu69	2.056	1_655	S
C46	C47	1.486	.	S
C46	C67	1.489	.	S
C47	O48	1.250	.	D
C47	C66	1.492	.	S
O48	Cu70	2.070	1_545	S
O49	C50	1.240	.	D
O49	Cu70	2.048	.	S
C50	C51	1.481	.	S
C50	C63	1.492	.	S
C51	O52	1.251	.	D
C51	C62	1.490	.	S
O52	Cu69	2.048	.	S
O53	C54	1.239	.	D
O53	Cu71	2.065	1_655	S
C54	C55	1.486	.	S
C54	C7	1.490	1_556	S
C55	O56	1.250	.	D
C55	C6	1.492	1_556	S
O56	Cu72	2.069	1_565	S
O57	C58	1.240	.	D
O57	Cu72	2.053	.	S
C58	C59	1.482	.	S
C58	C3	1.491	1_556	S
C59	O60	1.251	.	D
C59	C2	1.491	1_556	S
O60	Cu71	2.057	.	S
O61	C62	1.240	.	D
O61	Cu71	2.054	.	S

```
C62    C63     1.485   .     S
C63    O64     1.250   .     D
O64    Cu72    2.066   1_565 S
O65    C66     1.240   .     D
O65    Cu72    2.048   .     S
C66    C67     1.482   .     S
C67    O68     1.251   .     D
O68    Cu71    2.053   1_655 S
Cu69   O45     2.056   1_455 S
Cu69   O44     2.060   1_455 S
Cu70   O40     2.067   1_565 S
Cu70   O48     2.070   1_565 S
Cu71   O53     2.065   1_455 S
Cu71   O68     2.053   1_455 S
Cu72   O56     2.069   1_545 S
Cu72   O64     2.066   1_545 S
```

Copper squarate with one carbon dioxide molecule adsorbed

```
data_squarate+1co2
_audit_creation_date              2024-03-20
_audit_creation_method            'Materials Studio'
_symmetry_space_group_name_H-M    'P1'
_symmetry_Int_Tables_number       1
_symmetry_cell_setting            triclinic
loop_
_symmetry_equiv_pos_as_xyz
  x,y,z
_cell_length_a                    11.2119
_cell_length_b                    9.0707
_cell_length_c                    11.2086
_cell_angle_alpha                 89.2781
_cell_angle_beta                  116.2457
_cell_angle_gamma                 90.6069
loop_
_atom_site_label
_atom_site_type_symbol
_atom_site_fract_x
_atom_site_fract_y
_atom_site_fract_z
_atom_site_U_iso_or_equiv
_atom_site_adp_type
_atom_site_occupancy
O1     O     0.12338   0.24811   0.11157   0.00000  Uiso
1.00
C2     C     0.19186   0.25249   0.04998   0.00000  Uiso
1.00
C3     C     0.30730   0.16473   0.06059   0.00000  Uiso
1.00
O4     O     0.37139   0.06307   0.13838   0.00000  Uiso
1.00
O5     O     0.62164   0.74678   0.11529   0.00000  Uiso
1.00
C6     C     0.68960   0.75100   0.05283   0.00000  Uiso
1.00
C7     C     0.80472   0.66306   0.06335   0.00000  Uiso
1.00
```

O8	O	0.86934	0.56162	0.14085	0.00000	Uiso	1.00
O9	O	0.88610	0.24360	0.14676	0.00000	Uiso	1.00
C10	C	0.81351	0.24980	0.20386	0.00000	Uiso	1.00
C11	C	0.69304	0.16533	0.18716	0.00000	Uiso	1.00
O12	O	0.62795	0.06606	0.10830	0.00000	Uiso	1.00
O13	O	0.37727	0.74906	0.13693	0.00000	Uiso	1.00
C14	C	0.30310	0.75840	0.19222	0.00000	Uiso	1.00
C15	C	0.18316	0.67502	0.17728	0.00000	Uiso	1.00
O16	O	0.11950	0.57233	0.10152	0.00000	Uiso	1.00
O17	O	0.87531	0.75335	0.38230	0.00000	Uiso	1.00
C18	C	0.81179	0.75256	0.44896	0.00000	Uiso	1.00
C19	C	0.69801	0.84132	0.44127	0.00000	Uiso	1.00
O20	O	0.63013	0.93987	0.36175	0.00000	Uiso	1.00
O21	O	0.36434	0.24126	0.37547	0.00000	Uiso	1.00
C22	C	0.29924	0.23854	0.44103	0.00000	Uiso	1.00
C23	C	0.18615	0.32810	0.43337	0.00000	Uiso	1.00
O24	O	0.12017	0.42905	0.35609	0.00000	Uiso	1.00
O25	O	0.11380	0.75583	0.35449	0.00000	Uiso	1.00
C26	C	0.18030	0.75604	0.29069	0.00000	Uiso	1.00
C27	C	0.30103	0.83908	0.30638	0.00000	Uiso	1.00
O28	O	0.37386	0.92884	0.39179	0.00000	Uiso	1.00
O29	O	0.61007	0.25679	0.35085	0.00000	Uiso	1.00
C30	C	0.68518	0.25008	0.29649	0.00000	Uiso	1.00
C31	C	0.80626	0.33409	0.31430	0.00000	Uiso	1.00
O32	O	0.87125	0.43317	0.39378	0.00000	Uiso	1.00
Cu33	Cu	0.00320	0.40992	0.12807	0.00000	Uiso	1.00
Cu34	Cu	0.50306	0.91023	0.12821	0.00000	Uiso	1.00
Cu35	Cu	-0.00383	0.58980	0.37159	0.00000	Uiso	1.00

Cu36	Cu	0.49606	0.08929	0.37268	0.00000	Uiso 1.00
O37	O	0.12447	0.24662	0.61763	0.00000	Uiso 1.00
C38	C	0.18798	0.24741	0.55095	0.00000	Uiso 1.00
C39	C	0.30177	0.15864	0.55880	0.00000	Uiso 1.00
O40	O	0.36952	0.06014	0.63849	0.00000	Uiso 1.00
O41	O	0.63551	0.75875	0.62461	0.00000	Uiso 1.00
C42	C	0.70059	0.76139	0.55903	0.00000	Uiso 1.00
C43	C	0.81365	0.67178	0.56652	0.00000	Uiso 1.00
O44	O	0.87967	0.57071	0.64363	0.00000	Uiso 1.00
O45	O	0.88578	0.24414	0.64492	0.00000	Uiso 1.00
C46	C	0.81947	0.24401	0.70892	0.00000	Uiso 1.00
C47	C	0.69877	0.16100	0.69358	0.00000	Uiso 1.00
O48	O	0.62572	0.07114	0.60846	0.00000	Uiso 1.00
O49	O	0.38990	0.74298	0.64920	0.00000	Uiso 1.00
C50	C	0.31485	0.74983	0.70363	0.00000	Uiso 1.00
C51	C	0.19371	0.66604	0.68569	0.00000	Uiso 1.00
O52	O	0.12864	0.56712	0.60603	0.00000	Uiso 1.00
O53	O	0.87674	0.75183	0.88828	0.00000	Uiso 1.00
C54	C	0.80830	0.74747	0.94985	0.00000	Uiso 1.00
C55	C	0.69292	0.83530	0.93944	0.00000	Uiso 1.00
O56	O	0.62883	0.93707	0.86184	0.00000	Uiso 1.00
O57	O	0.37866	0.25323	0.88477	0.00000	Uiso 1.00
C58	C	0.31066	0.24895	0.94719	0.00000	Uiso 1.00
C59	C	0.19549	0.33686	0.93650	0.00000	Uiso 1.00
O60	O	0.13097	0.43828	0.85891	0.00000	Uiso 1.00
O61	O	0.11407	0.75658	0.85338	0.00000	Uiso 1.00
C62	C	0.18660	0.75031	0.79620	0.00000	Uiso 1.00
C63	C	0.30715	0.83460	0.81299	0.00000	Uiso 1.00

```
O64     O      0.37238   0.93377   0.89199   0.00000  Uiso
1.00
O65     O      0.62307   0.25118   0.86334   0.00000  Uiso
1.00
C66     C      0.69706   0.24181   0.80783   0.00000  Uiso
1.00
C67     C      0.81696   0.32511   0.82246   0.00000  Uiso
1.00
O68     O      0.88088   0.42777   0.89816   0.00000  Uiso
1.00
Cu69    Cu     0.00372   0.41012   0.62766   0.00000  Uiso
1.00
Cu70    Cu     0.50348   0.91055   0.62806   0.00000  Uiso
1.00
Cu71    Cu    -0.00265   0.59017   0.87147   0.00000  Uiso
1.00
Cu72    Cu     0.49742   0.08993   0.87257   0.00000  Uiso
1.00
C73     C      0.49973   0.50004   0.50000   0.00000  Uiso
1.00
O74     O      0.39847   0.55760   0.43766   0.00000  Uiso
1.00
O75     O      0.60099   0.44245   0.56233   0.00000  Uiso
1.00
loop_
_geom_bond_atom_site_label_1
_geom_bond_atom_site_label_2
_geom_bond_distance
_geom_bond_site_symmetry_2
_ccdc_geom_bond_type
O1      C2       1.239    .      D
O1      Cu33     2.067    .      S
C2      C3       1.485    .      S
C2      C59      1.492    1_554  S
C3      O4       1.252    .      D
C3      C58      1.489    1_554  S
O4      Cu34     2.076    1_545  S
O5      C6       1.242    .      D
O5      Cu34     2.050    .      S
C6      C7       1.484    .      S
C6      C55      1.489    1_554  S
C7      O8       1.250    .      D
C7      C54      1.492    1_554  S
O8      Cu33     2.099    1_655  S
O9      C10      1.240    .      D
O9      Cu33     2.058    1_655  S
C10     C11      1.485    .      S
C10     C31      1.496    .      S
C11     O12      1.249    .      D
C11     C30      1.490    .      S
O12     Cu34     2.058    1_545  S
O13     C14      1.242    .      D
O13     Cu34     2.052    .      S
C14     C15      1.480    .      S
C14     C27      1.491    .      S
C15     O16      1.252    .      D
```

C15	C26	1.489	.	S
O16	Cu33	2.062	.	S
O17	C18	1.239	.	D
O17	Cu35	2.060	1_655	S
C18	C19	1.485	.	S
C18	C43	1.492	.	S
C19	O20	1.252	.	D
C19	C42	1.487	.	S
O20	Cu36	2.079	1_565	S
O21	C22	1.244	.	D
O21	Cu36	2.041	.	S
C22	C23	1.483	.	S
C22	C39	1.487	.	S
C23	O24	1.250	.	D
C23	C38	1.492	.	S
O24	Cu35	2.086	.	S
O25	C26	1.241	.	D
O25	Cu35	2.054	.	S
C26	C27	1.484	.	S
C27	O28	1.249	.	D
O28	Cu36	2.062	1_565	S
O29	C30	1.241	.	D
O29	Cu36	2.056	.	S
C30	C31	1.485	.	S
C31	O32	1.251	.	D
O32	Cu35	2.073	1_655	S
Cu33	O9	2.058	1_455	S
Cu33	O8	2.099	1_455	S
Cu34	O4	2.076	1_565	S
Cu34	O12	2.058	1_565	S
Cu35	O17	2.060	1_455	S
Cu35	O32	2.073	1_455	S
Cu36	O20	2.079	1_545	S
Cu36	O28	2.062	1_545	S
O37	C38	1.239	.	D
O37	Cu69	2.056	.	S
C38	C39	1.485	.	S
C39	O40	1.252	.	D
O40	Cu70	2.077	1_545	S
O41	C42	1.244	.	D
O41	Cu70	2.045	.	S
C42	C43	1.483	.	S
C43	O44	1.251	.	D
O44	Cu69	2.088	1_655	S
O45	C46	1.240	.	D
O45	Cu69	2.057	1_655	S
C46	C47	1.485	.	S
C46	C67	1.489	.	S
C47	O48	1.249	.	D
C47	C66	1.491	.	S
O48	Cu70	2.065	1_545	S
O49	C50	1.241	.	D
O49	Cu70	2.051	.	S
C50	C51	1.484	.	S
C50	C63	1.490	.	S
C51	O52	1.251	.	D

```
C51    C62    1.496   .     S
O52    Cu69   2.073   .     S
O53    C54    1.239   .     D
O53    Cu71   2.071   1_655 S
C54    C55    1.486   .     S
C54    C7     1.492   1_556 S
C55    O56    1.251   .     D
C55    C6     1.489   1_556 S
O56    Cu72   2.077   1_565 S
O57    C58    1.242   .     D
O57    Cu72   2.048   .     S
C58    C59    1.483   .     S
C58    C3     1.489   1_556 S
C59    O60    1.250   .     D
C59    C2     1.492   1_556 S
O60    Cu71   2.097   .     S
O61    C62    1.240   .     D
O61    Cu71   2.054   .     S
C62    C63    1.484   .     S
C63    O64    1.250   .     D
O64    Cu72   2.059   1_565 S
O65    C66    1.242   .     D
O65    Cu72   2.053   .     S
C66    C67    1.480   .     S
C67    O68    1.252   .     D
O68    Cu71   2.064   1_655 S
Cu69   O45    2.057   1_455 S
Cu69   O44    2.088   1_455 S
Cu70   O40    2.077   1_565 S
Cu70   O48    2.065   1_565 S
Cu71   O53    2.071   1_455 S
Cu71   O68    2.064   1_455 S
Cu72   O56    2.077   1_545 S
Cu72   O64    2.059   1_545 S
C73    O74    1.163   .     D
C73    O75    1.163   .     D
```

Copper squarate with one water molecule adsorbed

```
data_squarate+1water
_audit_creation_date              2024-03-20
_audit_creation_method            'Materials Studio'
_symmetry_space_group_name_H-M    'P1'
_symmetry_Int_Tables_number       1
_symmetry_cell_setting            triclinic
loop_
_symmetry_equiv_pos_as_xyz
  x,y,z
_cell_length_a                    11.2143
_cell_length_b                    9.0155
_cell_length_c                    11.1874
_cell_angle_alpha                 90.2791
_cell_angle_beta                  116.1175
_cell_angle_gamma                 89.7141
loop_
_atom_site_label
```

```
_atom_site_type_symbol
_atom_site_fract_x
_atom_site_fract_y
_atom_site_fract_z
_atom_site_U_iso_or_equiv
_atom_site_adp_type
_atom_site_occupancy
O1      O      0.12294   0.24942   0.11052   0.00000  Uiso
1.00
C2      C      0.19226   0.25605   0.04967   0.00000  Uiso
1.00
C3      C      0.31143   0.17200   0.06319   0.00000  Uiso
1.00
O4      O      0.37893   0.07349   0.14278   0.00000  Uiso
1.00
O5      O      0.60921   0.73783   0.10302   0.00000  Uiso
1.00
C6      C      0.68306   0.74494   0.04660   0.00000  Uiso
1.00
C7      C      0.80394   0.66203   0.06312   0.00000  Uiso
1.00
O8      O      0.87039   0.56293   0.14284   0.00000  Uiso
1.00
O9      O      0.88222   0.24307   0.13674   0.00000  Uiso
1.00
C10     C      0.81441   0.24676   0.19919   0.00000  Uiso
1.00
C11     C      0.69795   0.15926   0.18835   0.00000  Uiso
1.00
O12     O      0.63258   0.05850   0.10998   0.00000  Uiso
1.00
O13     O      0.36276   0.75787   0.11995   0.00000  Uiso
1.00
C14     C      0.30110   0.75972   0.18888   0.00000  Uiso
1.00
C15     C      0.18789   0.67089   0.18218   0.00000  Uiso
1.00
O16     O      0.12189   0.56974   0.10479   0.00000  Uiso
1.00
O17     O      0.88157   0.75473   0.38654   0.00000  Uiso
1.00
C18     C      0.81428   0.75031   0.44992   0.00000  Uiso
1.00
C19     C      0.69443   0.83285   0.43623   0.00000  Uiso
1.00
O20     O      0.62385   0.92740   0.35392   0.00000  Uiso
1.00
O21     O      0.38583   0.25705   0.38878   0.00000  Uiso
1.00
C22     C      0.31542   0.25164   0.44861   0.00000  Uiso
1.00
C23     C      0.19598   0.33563   0.43485   0.00000  Uiso
1.00
O24     O      0.12847   0.43430   0.35529   0.00000  Uiso
1.00
```

O25	O	0.12475	0.75451	0.36466	0.00000	Uiso	1.00
C26	C	0.18943	0.75234	0.29878	0.00000	Uiso	1.00
C27	C	0.30466	0.83966	0.30738	0.00000	Uiso	1.00
O28	O	0.37283	0.93722	0.38785	0.00000	Uiso	1.00
O29	O	0.62503	0.25069	0.36213	0.00000	Uiso	1.00
C30	C	0.69518	0.24427	0.30102	0.00000	Uiso	1.00
C31	C	0.81107	0.33135	0.31283	0.00000	Uiso	1.00
O32	O	0.87576	0.43278	0.39069	0.00000	Uiso	1.00
Cu33	Cu	0.00048	0.41022	0.12533	0.00000	Uiso	1.00
Cu34	Cu	0.49569	0.91034	0.11883	0.00000	Uiso	1.00
Cu35	Cu	0.00222	0.59060	0.37349	0.00000	Uiso	1.00
Cu36	Cu	0.50303	0.08927	0.37432	0.00000	Uiso	1.00
O37	O	0.12373	0.24476	0.60892	0.00000	Uiso	1.00
C38	C	0.19349	0.25155	0.54847	0.00000	Uiso	1.00
C39	C	0.31291	0.16791	0.56210	0.00000	Uiso	1.00
O40	O	0.38076	0.06978	0.64196	0.00000	Uiso	1.00
O41	O	0.62617	0.74461	0.61367	0.00000	Uiso	1.00
C42	C	0.69460	0.75108	0.55214	0.00000	Uiso	1.00
C43	C	0.81405	0.66823	0.56482	0.00000	Uiso	1.00
O44	O	0.88131	0.56864	0.64357	0.00000	Uiso	1.00
O45	O	0.88176	0.24795	0.63682	0.00000	Uiso	1.00
C46	C	0.81317	0.25154	0.69868	0.00000	Uiso	1.00
C47	C	0.69735	0.16320	0.68724	0.00000	Uiso	1.00
O48	O	0.63300	0.06218	0.60837	0.00000	Uiso	1.00
O49	O	0.38126	0.74903	0.63680	0.00000	Uiso	1.00
C50	C	0.31050	0.75487	0.69627	0.00000	Uiso	1.00
C51	C	0.19406	0.66690	0.68343	0.00000	Uiso	1.00
O52	O	0.13162	0.56419	0.60599	0.00000	Uiso	1.00

```
O53    O     0.88138   0.75572   0.89359   0.00000  Uiso
1.00
C54    C     0.80951   0.74758   0.95159   0.00000  Uiso
1.00
C55    C     0.68903   0.83061   0.93586   0.00000  Uiso
1.00
O56    O     0.62311   0.92995   0.85619   0.00000  Uiso
1.00
O57    O     0.38475   0.26027   0.88996   0.00000  Uiso
1.00
C58    C     0.31409   0.25505   0.94959   0.00000  Uiso
1.00
C59    C     0.19465   0.33915   0.93557   0.00000  Uiso
1.00
O60    O     0.12702   0.43712   0.85517   0.00000  Uiso
1.00
O61    O     0.11568   0.75769   0.85336   0.00000  Uiso
1.00
C62    C     0.18671   0.75267   0.79433   0.00000  Uiso
1.00
C63    C     0.30344   0.84057   0.80728   0.00000  Uiso
1.00
O64    O     0.36637   0.94272   0.88526   0.00000  Uiso
1.00
O65    O     0.62598   0.24984   0.86397   0.00000  Uiso
1.00
C66    C     0.69369   0.24607   0.80116   0.00000  Uiso
1.00
C67    C     0.80919   0.33464   0.81242   0.00000  Uiso
1.00
O68    O     0.87330   0.43578   0.89091   0.00000  Uiso
1.00
Cu69   Cu    0.00508   0.40962   0.62436   0.00000  Uiso
1.00
Cu70   Cu    0.50568   0.90933   0.62647   0.00000  Uiso
1.00
Cu71   Cu   -0.00125   0.59146   0.87226   0.00000  Uiso
1.00
Cu72   Cu    0.50019   0.09084   0.87322   0.00000  Uiso
1.00
H73    H     0.52084   0.57019   1.24177   0.00000  Uiso
1.00
O74    O     0.52383   0.55285   1.32779   0.00000  Uiso
1.00
H75    H     0.54638   0.44920   1.34660   0.00000  Uiso
1.00
loop_
_geom_bond_atom_site_label_1
_geom_bond_atom_site_label_2
_geom_bond_distance
_geom_bond_site_symmetry_2
_ccdc_geom_bond_type
O1     C2      1.240   .     D
O1     Cu33    2.048   .     S
C2     C3      1.482   .     S
C2     C59     1.494   1_554 S
```

C3	O4	1.250	.	D
C3	C58	1.490	1_554	S
O4	Cu34	2.058	1_545	S
O5	C6	1.245	.	D
O5	Cu34	2.061	.	S
C6	C7	1.484	.	S
C6	C55	1.490	1_554	S
C7	O8	1.251	.	D
C7	C54	1.495	1_554	S
O8	Cu33	2.071	1_655	S
O9	C10	1.239	.	D
O9	Cu33	2.054	1_655	S
C10	C11	1.487	.	S
C10	C31	1.494	.	S
C11	O12	1.251	.	D
C11	C30	1.484	.	S
O12	Cu34	2.075	1_545	S
O13	C14	1.242	.	D
O13	Cu34	2.037	.	S
C14	C15	1.478	.	S
C14	C27	1.491	.	S
C15	O16	1.251	.	D
C15	C26	1.487	.	S
O16	Cu33	2.067	.	S
O17	C18	1.243	.	D
O17	Cu35	2.048	1_655	S
C18	C19	1.481	.	S
C18	C43	1.487	.	S
C19	O20	1.249	.	D
C19	C42	1.494	.	S
O20	Cu36	2.066	1_565	S
O21	C22	1.241	.	D
O21	Cu36	2.051	.	S
C22	C23	1.484	.	S
C22	C39	1.491	.	S
C23	O24	1.251	.	D
C23	C38	1.494	.	S
O24	Cu35	2.065	.	S
O25	C26	1.241	.	D
O25	Cu35	2.055	.	S
C26	C27	1.483	.	S
C27	O28	1.250	.	D
O28	Cu36	2.062	1_565	S
O29	C30	1.250	.	D
O29	Cu36	2.048	.	S
C30	C31	1.477	.	S
C31	O32	1.250	.	D
O32	Cu35	2.082	1_655	S
Cu33	O9	2.054	1_455	S
Cu33	O8	2.071	1_455	S
Cu34	O4	2.058	1_565	S
Cu34	O12	2.075	1_565	S
Cu35	O17	2.048	1_455	S
Cu35	O32	2.082	1_455	S
Cu36	O20	2.066	1_545	S
Cu36	O28	2.062	1_545	S

O37	C38	1.241	.	D
O37	Cu69	2.047	.	S
C38	C39	1.482	.	S
C39	O40	1.250	.	D
O40	Cu70	2.068	1_545	S
O41	C42	1.238	.	D
O41	Cu70	2.049	.	S
C42	C43	1.482	.	S
C43	O44	1.252	.	D
O44	Cu69	2.066	1_655	S
O45	C46	1.241	.	D
O45	Cu69	2.061	1_655	S
C46	C47	1.484	.	S
C46	C67	1.491	.	S
C47	O48	1.252	.	D
C47	C66	1.490	.	S
O48	Cu70	2.063	1_545	S
O49	C50	1.241	.	D
O49	Cu70	2.052	.	S
C50	C51	1.483	.	S
C50	C63	1.491	.	S
C51	O52	1.250	.	D
C51	C62	1.491	.	S
O52	Cu69	2.068	.	S
O53	C54	1.240	.	D
O53	Cu71	2.058	1_655	S
C54	C55	1.483	.	S
C54	C7	1.495	1_556	S
C55	O56	1.251	.	D
C55	C6	1.490	1_556	S
O56	Cu72	2.061	1_565	S
O57	C58	1.241	.	D
O57	Cu72	2.058	.	S
C58	C59	1.483	.	S
C58	C3	1.490	1_556	S
C59	O60	1.251	.	D
C59	C2	1.494	1_556	S
O60	Cu71	2.064	.	S
O61	C62	1.239	.	D
O61	Cu71	2.068	.	S
C62	C63	1.485	.	S
C63	O64	1.251	.	D
O64	Cu72	2.062	1_565	S
O65	C66	1.241	.	D
O65	Cu72	2.051	.	S
C66	C67	1.482	.	S
C67	O68	1.250	.	D
O68	Cu71	2.067	1_655	S
Cu69	O45	2.061	1_455	S
Cu69	O44	2.066	1_455	S
Cu70	O40	2.068	1_565	S
Cu70	O48	2.063	1_565	S
Cu71	O53	2.058	1_455	S
Cu71	O68	2.067	1_455	S
Cu72	O56	2.061	1_545	S
Cu72	O64	2.062	1_545	S

H73	O74	0.962	.	S
O74	H75	0.966	.	S
